# Identification and Characterization of the RouenBd1987 *Babesia divergens* Rhopty-Associated Protein 1

**DOI:** 10.1371/journal.pone.0107727

**Published:** 2014-09-16

**Authors:** Marilis Rodriguez, Andy Alhassan, Rosalynn L. Ord, Jeny R. Cursino-Santos, Manpreet Singh, Jeremy Gray, Cheryl A. Lobo

**Affiliations:** 1 Department of Blood-Borne Parasites, New York Blood Center, New York, New York, United States of America; 2 University College Dublin School of Biology and Environmental Science, Dublin, Republic of Ireland; Bernhard Nocht Institute for Tropical Medicine, Germany

## Abstract

Human babesiosis is caused by one of several babesial species transmitted by ixodid ticks that have distinct geographical distributions based on the presence of competent animal hosts. The pathology of babesiosis, like malaria, is a consequence of the parasitaemia which develops through the cyclical replication of *Babesia* parasites in a patient's red blood cells, though symptoms typically are nonspecific. We have identified the gene encoding Rhoptry-Associated Protein −1 (RAP-1) from a human isolate of *B. divergens*, Rouen1987 and characterized its protein product at the molecular and cellular level. Consistent with other *Babesia* RAP-1 homologues, BdRAP-1 is expressed as a 46 kDa protein in the parasite rhoptries, suggesting a possible role in red cell invasion. Native BdRAP-1 binds to an unidentified red cell receptor(s) that appears to be non-sialylated and non-proteinacious in nature, but we do not find significant reduction in growth with anti-rRAP1 antibodies *in vitro*, highlighting the possibility the *B. divergens* is able to use alternative pathways for invasion, or there is an alternative, complementary, role for BdRAP-1 during the invasion process. As it is the parasite's ability to recognize and then invade host cells which is central to clinical disease, characterising and understanding the role of *Babesia*-derived proteins involved in these steps are of great interest for the development of an effective prophylaxis.

## Introduction

The phylum Apicomplexa consists of protozoan parasites, among which are a number of etiological agents of medical and veterinary importance, including *Plasmodium*, *Toxoplasma*, *Babesia*, *Neospora*, *Cryptosporidium*, *Eimeria* and *Theileria*. They are defined by a common set of apically located secretory organelles that are required for host cell invasion [1]–[3]. Although the host cell range of Apicomplexan parasites is large and varied, the mechanism of host cell entry is thought to be conserved [4].

The genus *Babesia* comprises many species of parasites that invade RBCs of many different vertebrate hosts [5]. They are transmitted by their tick vectors during the taking of a blood meal from the vertebrate host [5], [6]. In the last 50 years babesiosis has emerged as a major public health concern due to the expansion of the geographic range of the vectors, ixodid ticks [7], [8]. Additionally, fatality rates average 30% to 45% in susceptible hosts, which include older subjects, new-born infants, and people that are immune-compromised [9]. As a consequence, since 2011, babesiosis is now a nationally notifiable disease in 18 states in the United States [10]. Human babesiosis is caused by one of several babesial species that have distinct geographical distributions based on the presence of competent animal hosts [11]. In Europe, babesiosis in man is caused by the bovine pathogen *B. divergens* [12], [13]; in North America, it is caused predominantly by *B. microti* [14], a rodent borne parasite.

The pathology of babesiosis, like malaria, is a consequence of the parasitaemia which develops through the cyclical replication of *Babesia* parasites in a patient's RBC's, though symptoms typically are nonspecific (fever, headache, and myalgia) [15]. The parasite's ability to first recognize and then invade host RBCs is central to the disease process, and thus *Babesia*-derived proteins involved in these recognition and invasion steps are of great interest for the development of an effective prophylaxis.

Rhoptries are club-shaped organelles located at the apical end of the invading merozoite. They are divided into two distinct intra-organelle compartments, the posterior bulb and the more anterior duct (neck) through which rhoptry proteins are secreted [16], [17]. Rhoptry contents include both protein and lipid components, which assemble to form membrane-like structures. Proteins derived from the rhoptries are crucial for the invasion and survival of these parasites. Among these proteins in malaria, two rhoptry-associated protein complexes are considered attractive anti-parasitic targets: the high molecular weight (HMW or RhopH complex) and the low molecular weight (LMW) complexes; the first one containing the RhopH-CLAG proteins and the second one consisting of the rhoptry-associated proteins (RAP)-1, RAP-2 and RAP-3 [16], [18]. Homologs of these two sets of rhoptry proteins have not been identified in any of the Babesia species. However a number of rhoptry molecules classified as RAP-1 and RAP-2 have been characterized in some of the veterinary parasite species.

There is a brief report on the gene sequence of a RAP-1 in *B. divergens* [19] isolated from a bovine source, although there are no further details on the characterization of this antigen. As *B. divergens* is an important human zoonosis, it is prudent to fully assess genes responsible for RBC invasion from parasites that result in human infection to aid the most effective interventions. We have identified and characterised a Rhoptry Associated Protein −1 (RAP-1) homolog of BdRouen1987 isolated from, a human infection, and provide additional support for this antigen role in invasion.

## Materials and Methods

### Animal Protocol Work and Ethics Statement

Cattle and gerbil sera were produced in 1995 under license in the Republic of Ireland (License number B100/702, Department of Health, Cruelty to Animal Act, 1876 (European Directive 86/609/EC) as part of studies on vaccination against bovine babesiosis. The license provided permitted the use of infesting with ticks, infecting with *Babesia divergens* by intraperitoneal injection, taking of blood spots from the tail for blood smears and bleeding while under halothane anaesthesia without recovery (euthanasia). As no formal ethics (IACUC) committees for animal experimentation existed in Ireland at the time (1995), the conditions of the licence were approved at the University College Dublin by both the Director of the Biomedical Facility (the departmental animal facility) and the Dean of the Faculty of Veterinary Sciences.

Briefly, yearling cattle housed on the University College Dublin farm were inoculated subcutaneously behind the left shoulder with 1×10^7^ gerbil (*Meriones unguiculatus*) RBCs infected with the TL strain of *B. divergens* and suspended in 60% RPMI 1640 in HEPES with added L-glutamine and 40% fetal calf serum. Four weeks later approximately 100 mL of venous blood were taken by syringe from the jugular vein, and antibody levels of 1∶256 titre in the resulting sera determined by IFA. The sera were then stored at −70°C.

Gerbils were housed at the Biomedical Facility, University College Dublin and sera were prepared by intraperitoneal inoculation of a group of 8-week old gerbils with PBS-suspended 1×10^2^ erythrocytes infected with *B. divergens* (DR strain). Three weeks later the gerbils in which transient parasitaemia had been observed were challenged with 1×10^3^ infected erythrocytes subdermally and then after a further three weeks with 1×10^7^ infected erythrocytes. After a further 4 weeks, all gerbils were bled by cardiac puncture under halothane anaesthesia and sacrificed after bleeding by intraperitoneal injection of sodium pentobarbitone formulated for euthanasia, and the resulting sera stored at −70°C.

*Babesia divergens* strain BdRouen1987 was first isolated from a French patient in 1986 [20] and propagated in culture, and has been used by many laboratories as the reference strain for *B. divergens* studies [21]–[25].

### Parasite propagation

Asexual erythrocytic cultures of *B. divergens* (BdRouen1987 isolated from a French patient) [20] were maintained *in vitro* in human A^+^ blood using RPMI 1640 medium (Life Technologies Corporation, Carlsbad, CA) supplemented with 10% human serum and sodium bicarbonate solution 7.5% (w/v) (Life Technologies Corporation, Carlsbad, CA). Cells were cultured at 37°C in 90% C02, 5% nitrogen and 5% oxygen.

### Identification and confirmation of the cDNA and gDNA Bdrap-1 sequences

The Bdrap-1 sequence was obtained by initially screening a *B. divergens* cDNA library [22] by PCR with the universal primers, T3 and T7, in various combinations with degenerate primers based upon published rap-1 genes of alternative *Babesia* spp. Amplified PCR products representing partial Bdrap-1 sequences were separated on 1% agarose gels, cleaned using QIAquick gel extraction kit (Qiagen), cloned into TOPO TA vector (Life Technologies Corporation, Carlsbad, CA), and transformed into TOP10 *Echerichia coli* strain (Life Technologies Corporation, Carlsbad, CA) prior to sequencing. Partial Bdrap-1 sequences obtained were aligned using ClustalW (http://www.ebi.ac.uk/Tools/msa/clustalw2/) to design specific Bdrap-1 primers. Using various combinations of the T3 and T7 universal and Bdrap-1 specific primers, the full length sequence of the cDNA Bdrap-1 gene was obtained using the same amplification, and cloning strategy as above. The genomic sequence of the Bdrap-1 gene was obtained by amplifying 100 ng genomic *B. divergens* DNA (prepared as described in [25]) using specific primers Bdrap-1F1 (5′-GCTTTCGAAGCTTATTGACTCT-3′) and Bdrap-1R1 (5′ GTTAAAACCTTAGGGATAAATTGGTA 3′). The PCR product was separated on 1% agarose gel and cleaned using QIAquick gel extraction kit (Qiagen, Hilden, Germany), cloned into TOPO TA vector (Life Technologies Corporation, Carlsbad, CA), and transformed into TOP10 *Echerichia coli* strain (Life Technologies Corporation, Carlsbad, CA) prior to sequencing.

### Protein Expression and purification and rabbit antiserum production

Genomic *B. divergens* DNA encoding residues Val^32^ to Met^379^ of the BdRAP-1 protein was amplified using the modified primers Bdrap-1F2 (5′ CGCGGATCCGTGGCTCTTGGCGAG 3′), containing *BamH*I restriction site, and Bdrap-1R2 (5′ CCGCTCGAGTCAGAGTCCATGCCTGTA 3′), containing *Xho*I restriction site and stop codon, using SuperMix kit (Life Technologies Corporation, Carlsbad, CA). Amplified products were cloned into the expression vector, pET28(a) vector (Life Technologies Corporation, Carlsbad, CA) according to the manufacturer's instructions. *E. coli* strain BL21 was transformed with pET28a-Bdrap1 in 250 mL of SOB medium containing 100 µg/mL Ampicillin (Sigma-Aldrich, St Louis, MO) and inoculated with 1 mL of fresh overnight culture and grown at 37°C to A_600_ = 0.6 prior to induction with 0.2 isopropyl-β-D-thiogalactopyranoside. After 4 h of induction, recombinant BdRAP-1 protein was purified from bacterial cells using Ni-NTA Buffer kit as per manufactures instructions (EMD Millipore Corporation, Billerica, MA) supplemented with a protease inhibitor cocktail (Sigma-Aldrich, St Louis, MO). The insoluble material was removed by centrifugation and the soluble fraction was used to purify the rBdRAP-1 protein. Protein concentration was determined by the Bradfords Dye (Bio-Rad Laboratories, Hercules, CA). Polyclonal serum was then raised against the rBdRAP-1 in rabbits (Strategic Diagnostics Inc., Newark, DE).

### Immunoprecipitation

Freshly cultured parasites were washed and resuspended in methionine-free RPMI 1640 (MP Biomedicals Inc., Aurora, OH). 200 µCi/mL of [^35^S] methionine/cysteine (PerkinElmer, Boston, MA) was added and parasites were incubated at 37°C for 2 h. Parasites were lysed in NETT buffer [10 mM Tris pH 7.5, 150 mM NaCl, 0.5 mM EDTA, 0.1% Triton X-100] using a protease inhibitor cocktail (Sigma-Aldrich, St Louis, MO, USA)) and centrifuged to collect the supernatant. Lysates were pre-cleared with Protein G-Sepharose beads (GE Healthcare, Waukesha, WI) before antibody addition. Protein G-Sepharose beads were added and washed extensively with NETTS (10 mM Tris, pH 7.5, 500 mM, NaCl, 5 mM EDTA and 0.1% Triton X-100) and NETT buffers. Protein was eluted from the beads using elution buffer [10 mM Tris pH 7.5, 150 mM NaCl, 5 mM EDTA], boiled and run on SDS-PAGE gel. Gels were fixed with fixing solution [25% Isopropanol alcohol; 10% Acetic Acid] for 30 min and enhanced with Amplify™ fluorographic solution (GE Healthcare, Waukesha, WI) for 45 min, dried under vacuum, and exposed to X-ray film for autoradiography.

### Immunofluorescence assay (IFA)

Cultured parasites with a high parasitaemia (∼70%) or purified free merozoites were prepared for immunofluorescence (IFA). The parasites were smeared on glass slides and fixed in cold 90% acetone/10% methanol. The slides were incubated for 30 min with purified anti-rBdRAP-1 antibodies diluted 1∶200 in 1× PBS/1%BSA. The slides were washed three times in 1× PBS and incubated for 30 min with a polyclonal anti-rabbit immunoglobulin (Dako, Produktionsvej Denmark) diluted 1∶200 in 1× PBS/1% BSA. The slides were washed again three times in 1× PBS, rinsed once in distilled water, and mounted using Vectashield with DAPI solution for microscopy (Vector laboratories Inc., Burlingame, CA).

### Electron microscopy

Cultured parasites and free merozoites were fixed with 1% paraformaldehyde and 0.1% glutaraldehyde in 0.1 M cacodylate buffer, for 1 h at 4°C, washed in 0.1 M buffer, pH 7.4 and treated with 50 mM ammonium chloride to quench the remaining aldehydes. The fixed infected RBCs and the free merozoites were than dehydrated and embedded in LR-White Resin (Electron Microscopy Sciences, Hatfield, PA). Thin sections of embedded parasites were mounted on parlodion covered nickel grids, blocked in 2% BSA and probed with purified anti-rBdRAP-1 antibodies overnight at 4°C, washed in buffer containing 1× BSA and Tween-20 and incubated with goat-anti-Rabbit IgG coupled with 6 nm gold particles (Electron Microscopy Sciences, Hatfield, PA) or Goat-anti-Mouse IgG coupled with 5 nm gold particles (GE Healthcare, Waukesha, WI). After staining with uranyl acetate, sections were observed using a Philips-410 electron microscope.

### RBC Binding assays

#### RBCs

Erythrocytes collected in 10% citrate-phosphate-dextrose were washed 3 times in 1× PBS and treated with 1.0 mg/mL of neuraminidase and chymotripson, and 0.1 and 1.0 mg/mL of trypsin as described [25] or treated with Proteinase K (Life Technologies Corporation, Carlsbad, CA) and papain (Sigma-Aldrich, St Louis, MO) as follows: 500 uL (packed cell volume) of washed erythrocytes were incubated with 0.5 mg/mL Proteinase K for 30 in at 37°C in a final volume of 4 mL with 1×PBS. 500 uL (pvc) of washed erythrocytes were incubated with 0.5 mg/mL Papain at r.t for 30 min in a total volume of 2 mL.

#### Parasite Supernatant

Freshly cultured parasites were washed and re-suspended in methionine-free RPMI 1640 (MP Biomedicals Inc., Aurora, OH). 200 µCi/mL of [^35^S] methionine/cysteine (PerkinElmer, Boston, MA) was added, and parasites were incubated at 37°C overnight. The supernatant was collected by centrifugation at 14,000 rpm for 15 min and a protease inhibitor mixture (Sigma-Aldrich, St Louis, MO) was added. The parasite supernatant was pre-cleared with protein G (GE Healthcare, Waukesha, WI) before to use.

#### Binding assay

An aliquot of 500 µL of labelled parasite supernatant, confirmed as having Bd-RAP-1 by immunoprecipitation, was mixed with 100 µL target erythrocytes (wild type or enzyme treated) for 30 min at r.t. The mixture was then spun at 6000 *g* for 1 min through sodium phthalate oil to remove unbound material. Oil and supernatant was aspirated, leaving the RBC/protein complexes. Cells were lysed in NETT [0.5% Triton-X100, 150 mM NaCl, 10 mM EDTA 50 mM Tris, pH 7.4] for 30 minutes at 4°C and spun at 14, 000 rpm for 5 min. The soluble extract containing the BdRAP1-receptor complex was immunoprecipitated with anti-rBdRAP1 antibodies. All experiments were repeated at least 3 times with identical pattern of binding observed.

### ELISAs

Microtiter ELISA plates (Costar, Corning Life Sciences, Corning, NY) were coated with 1 µg/mL of recombinant BdRAP-1 protein diluted in 0.05 M carbonate buffer, pH 9.6. After incubation overnight at 4°C, the plates were washed 5 times with 1× phosphate-buffered saline (PBS) with 0.05% Tween-20 (1× PBS-T) and blocked with blocking buffer (3% BSA in 1× PBS-T) for 1 h at 37°C. Serum samples, diluted in blocking buffer, were reacted with the bound antigens by incubating for 1 h at 37°C, in triplicate wells. Serum from the cows was diluted from 1∶200 to 1∶12,800, and serum from the gerbils was diluted 1∶200 to 1∶25,600. The bound antibodies in the cow sera were detected after incubation for 1 h at 37°C with goat anti-cow conjugated HRP at 1∶1000 (Abcam, Cambridge, MA) diluted in blocking buffer; antibodies in the gerbil sera were detected after incubation for 1 h at 37°C with goat anti-rat conjugated HRP (Pierce Antibody Products, Thermo Fisher Scientific Inc, Rockford, IL) diluted 1∶10,000 in blocking buffer. Tetramethylbenzidine (Sigma, St Louis, MO) was used as the substrate for all ELISAs for 30 min. Sulphuric acid (2M) was used to stop the substrate reaction, and the optical density (OD) was read at 450 nm immediately on a SpectraMax 190 ELISA Reader (Molecular Devices, Sunnyvale, CA).

### In vitro growth inhibition assays (GIA)

Total preimmune rabbit (PI) and BdRAP-1 IgGs were purified using protein-G Sepharose (GE Healthcare, Waukesha, WI) with IgG binding and elution buffers (Thermo Fisher Scientific, Rockford, IL) according to the manufacturer's recommendation and dialyzed with DPBS. Purified pre-immune IgG (Sigma-Aldrich, St Louis, MO) and rBdRAP1 were added to pre-warmed medium and 5% haematocrit of RBCs at ∼4% parasitaemia. The final concentration of antibodies in the GIA was 2 mg/mL in a 500 uL final volume. Each antibody was tested in triplicate. The culture medium was removed at 24 h and replaced with fresh medium, 10% human serum, and purified IgG to a final concentration of 2 mg/mL. Samples were incubated at 37°C with 90% C02, 5% nitrogen, 5% oxygen. Smears were made at 0 h, 12 h, 24 h, 36 h. The slides were fixed in 100% methanol and stained with Giemsa (Sigma-Aldrich, St Louis, MO). Parasitaemia was determined after counting the total number of intracellular parasites present in 1×10^4^ RBC at ×100 magnification using a Nikon Eclipse E 600 microscope, and the invasion inhibition with respect to the controls was determined for each antibody tested.

## Results and Discussion

### Cloning of a RAP-1 homolog (BdRAP-1) of *B. divergensRouen1987*

The *B. divergens* genome has not yet been sequenced. Although a *B. divergens* RAP-1 sequence from a bovine infection has been identified [19], no BdRAP-1 homolog from a human infection has been identified, and it is possible that sequence polymorphisms may exist between strains that have alternative host preferences to enable specific host cell interactions. To clone the gene encoding BdRAP-1, a *B. divergens* cDNA library, created from material obtained from a strain isolated from a human *B. divergens* infection and maintained in culture [20], [22], was screened by PCR with the universal primers, T3/T7, in conjunction with degenerate primers based upon homology within published rap-1 genes of alternative *Babesia* spp. A band of ∼1 Kb was obtained and sequenced. BLAST analysis identified this sequence as encoding a putative conserved domain corresponding to the RAP-1 superfamily, as well as high homology to the rap-1 gene from other *Babesia* spp. This sequence was used to design specific Bdrap-1 primers, which were used with the universal primers, to continue screening the cDNA library. Amplified fragments were cloned into TOPO vector and sequenced multiple times. Nucleotide sequences were aligned using ClustalW2 (http://www.ebi.ac.uk/Tools/msa/clustalw2/) and assembled into a contig of ∼1,300 bp with at least 3× sequence coverage across all nucleotides. This contig contained 1,255 bp of the full ORF encoding BdRAP-1, which has been submitted into GenBank (accession number KJ699101). Multiple clones containing the ORF were isolated and sequenced and all yielded identical sequence. PCR amplification of the Bdrap-1 gene from genomic DNA with specific primers derived from the full-length cDNA Bdrap-1 contig produced a single fragment of 1,225 bp (GenBank accession KJ699102). Cloning and sequencing showed the genomic Bdrap-1 gene to be 1,225 bp, the same length and nucleotide sequence as the cDNA Bdrap-1 contig, suggesting the absence of introns in the gene encoding BdRAP-1.

### Comparison of BdRAP-1 sequence with other Apicomplexan sequences

**Figure 1:**
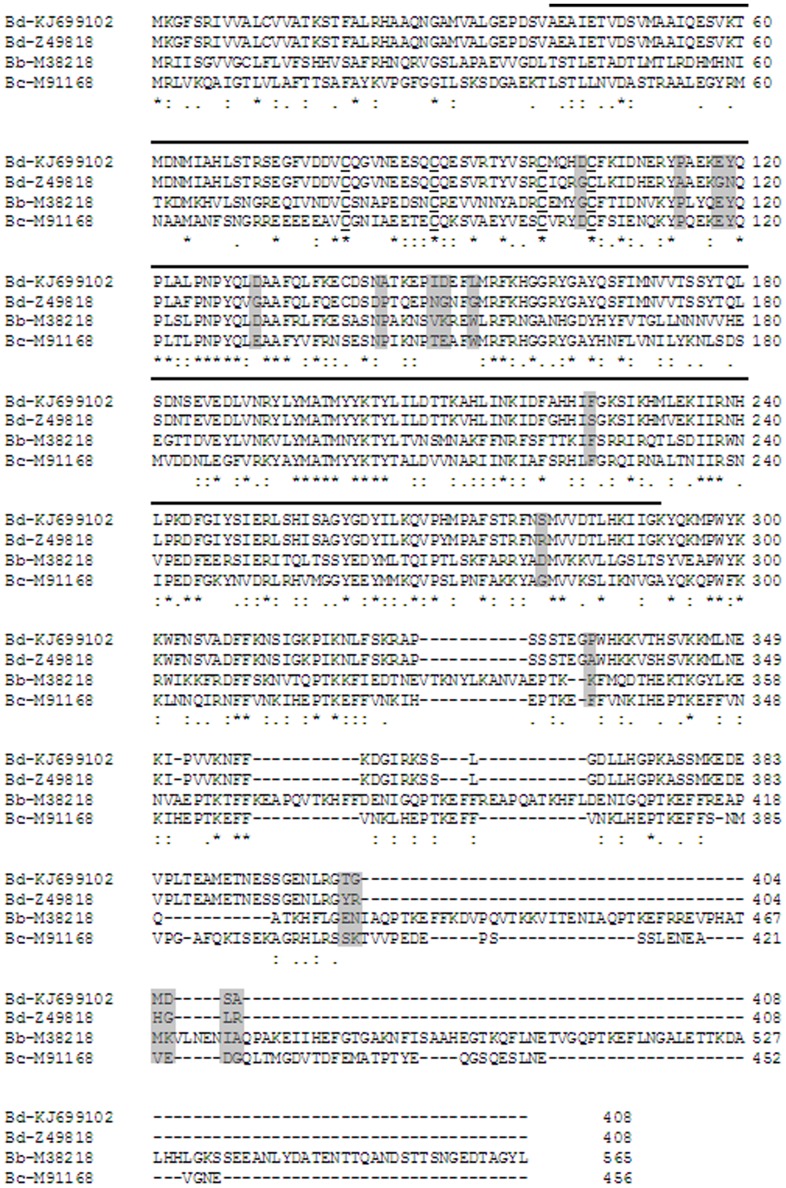
Multiple sequence alignment of BdRAP-1 sequences shows homology to other babesial RAP-1 proteins is within the N-terminal portion. Clustal analysis of RAP-1 sequences of *B. divergens* (human host source) (accession number KJ699102) identified in this manuscript, *B. divergens* (bovine host origin) (accession number Z49818), *B. bovis* (accession number M38218) and *B. canis* (accession number M91168) shows that homology is restricted to the N-terminus region, which corresponds to the location of the RAP-1 superfamily structure (indication above the sequence by a sold line from BdRAP-1 Ala^41^ to Gly^291^) as identified by BLAST analysis. The sites of non-synonymous differences between the BdRAP-1 proteins are shown in grey shading. Positions of identity are indicated by asterisk and similarity by dots, below the sequences. Four cysteine residues, at sites BdRAP-1 80, 89, 100, and 105 (bold underlined), are conserved across all species, and are also limited to the N-terminus region, further suggesting this portion of RAP-1 may contain the RBC binding site.

Conceptual translation of the 1,225 bp open reading frame resulted in prediction of a ∼46 kDa protein. A BLAST search with the full translated sequence identifies the region encompassing Ala^41^ to Gly^291^ as a member of the RAP-1 superfamily, as shown in Figure 1. The BdRAP-1 antigen we identified shows a 93% homology to a putative *B. divergens* RAP-1 protein isolated from a bovine infection [19], 47% identity to *B. canis* [26], and 33% identity to *B. bovis* [27], although there is very little identity to RAP-1 antigens of either *Plasmodium* or *Toxoplasma* spp (not shown). Sequence alignment with these RAP-1 proteins from other babesial species shows the N-terminal region that encompasses the predicted location of the RAP-1 superfamily in *B. divergens* is relatively conserved (∼40% homology) in both *B. canis* and *B. bovis*, and includes 4 conserved cysteine residues across the species, indicated by bold underline in Figure 1. There are19 non-synonymous polymorphisms between the BdRAP-1 amino acid sequence identified here from a human infection and the BdRAP-1 identified from a bovine infection (highlighted in grey in Figure 1). Although these polymorphisms appear spread throughout the amino acid sequence, it is interesting to note that all but one of these occur down-stream of the 4 conserved cysteine residues, suggesting the N-terminus region is the location of the RBC receptor binding site for all babesial RAP-1 antigens. It remains to be determined if the polymorphisms have a role in determining host cell preference for parasite invasion.

### Expression of BdRAP-1 as a hexa-His fusion protein and generation of antibodies

The sequence encoding Val^32^ to Met^379^ of BdRAP-1 was cloned into the expression vector pET28(a) and expressed in *E. coli* as a HIS fusion protein. The predicted mass of the recombinant product was ∼42 kDa, including the hexa-His-tag. The recombinant RAP-1 protein was purified from the soluble fraction and Coomassie staining shows the purified BdRAP-1 recombinant product is observed at ∼42 kDa (data not shown), as predicted from the gene sequence, and was used to immunize rabbits.

### BdRAP-1 is expressed as a 46 kDA protein in the erythrocytic parasite

**Figure 2:**
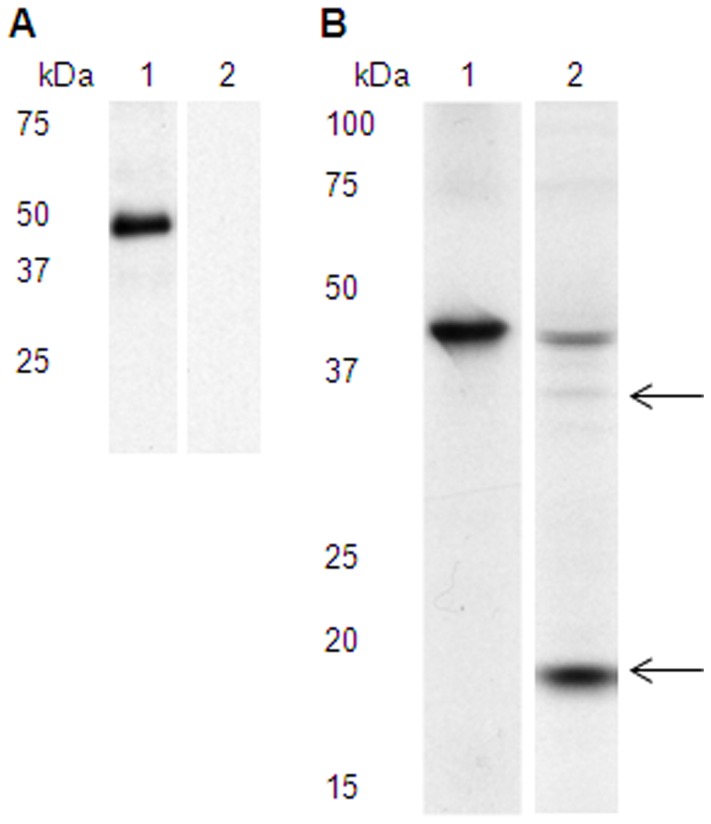
Anti-rBdRAP-1 antibodies identify a specific ∼46 kDa protein in parasite lysate. A, Immunoblotting of proteins from in vitro cultured *B. divergens* lysate showed anti-rBdRAP-1 antibodies (lane 1) are able to detect native BdRAP-1, as shown by the presence of a single band at ∼46 kDa. Pre-immune rabbit serum (lane 2) did not react with any native antigens. B, Immunoprecipitation with lysate from [^35^S] methionine/cysteine-labelled *B. divergens* cultures showed native BdRAP-1 is present in the culture pellet (lane 1), as observed by the presence of a single band at ∼46 kDa. BdRAP-1 is secreted into the culture supernatant as a processed product, as shown by the presence of an abundant ∼17 kDa product, and a much less abundant product of ∼34 kDa, in the culture supernatant (lane 2; indicated by arrows).

Immune sera from rabbits immunized with the purified rBdRAP-1 protein were used to characterize the native protein in the parasite. Immuno-blotting with native parasite lysate from infected cells showed a distinct single band of 46 kDa (Figure 2A, lane 1), but no products were detected with pre-immune sera (Figure 2A, lane 2). Immunoprecipitation (IP) with lysate from infected cells detected the same single band of 46 kDa, confirming the size of the native BdRAP-1 protein (Figure 2B, lane 1). Many rhoptry proteins undergo proteolytic processing during invasion. To investigate if BdRAP-1 was similarly processed, immunoprecipitations were carried out on spent culture supernatants from in vitro cultures of the parasite. These experiments revealed the 46 kDa native BdRAP-1 (Figure 2B, lane 2) along with a secondary, lower molecular-weight band of ∼20 kDa (Figure 2B, lane 2). These analyses suggest that the native BdRAP-1 undergoes proteolytic processing, similar to RAP-1 of *P. falciparum* [28], [29].

### BdRAP-1 localizes to the rhoptry organelles in the parasite

**Figure 3:**
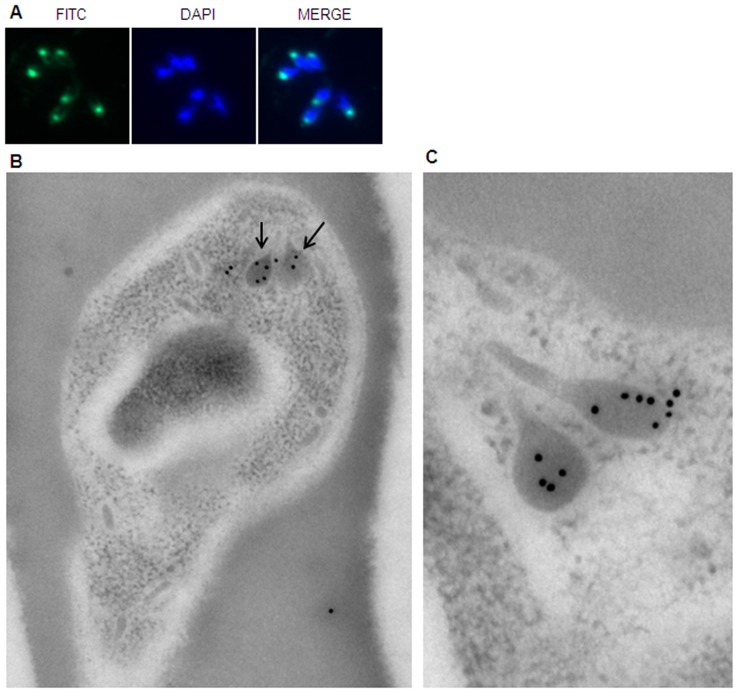
Native BdRAP-1 localised to apical complex. A, Immunofluorescence staining with anti-rBdRAP-1 antibodies (FITC) on fixed cells infected with *B. divergens* parasites (DAPI) clearly shows that BdRAP-1 is localised to the apical ends of all 4 parasites in this Maltese Cross form of the intracellular parasite (see Merged panel). B, Electron microscopy of a singly-infected RBC (direct magnification of 30,000×, print magnification of ∼50,000×) shows the location of the rhoptry organelles and the presence of BdRAP-1. C, An enlarged section (direct magnification 49,000×, print magnification of ∼140,000×) clearly shows localisation of native BdRAP-1 is within the bulb of the rhoptry organelle.

All the RAP-1 proteins that have been described so far have been localized to the apical, invasive end of the merozoite, and should be true of BdRAP-1 if it also plays a role in parasite entry. The subcellular localization of BdRAP-1 was determined by immunofluorescence assay (IFA) using anti-rBdRAP-1 rabbit antibodies. Figure 3A shows the staining pattern obtained on smears of asexual parasites demonstrating that BdRAP-1 is indeed located at the apical end of merozoites. The punctate staining in this region, wherein a single dot occasionally resolves into double foci, is a hallmark of rhoptry localization. We confirmed this specific localization of BdRAP-1 in rhoptries by immuno-electronmicroscopic analysis (IEM). The IEM was carried out on sections of parasite-infected RBC with anti-rBdRAP-1 antibodies. As can be seen in Figure 3B, discrete antibody reactivity was observed in these electron-dense organelles with the morphological characteristics of rhoptries (indicated by arrows in Figure 3B). Increased magification showed that BdRAP-1 is localized to the bulb of the rhoptries (Figure 3C), and thus may have an active role in host cell invasion.

### BdRAP-1 binds to erythrocytes via a trypsin-, chymotrypsin- and neuraminidase-resistant RBC receptor

**Figure 4:**
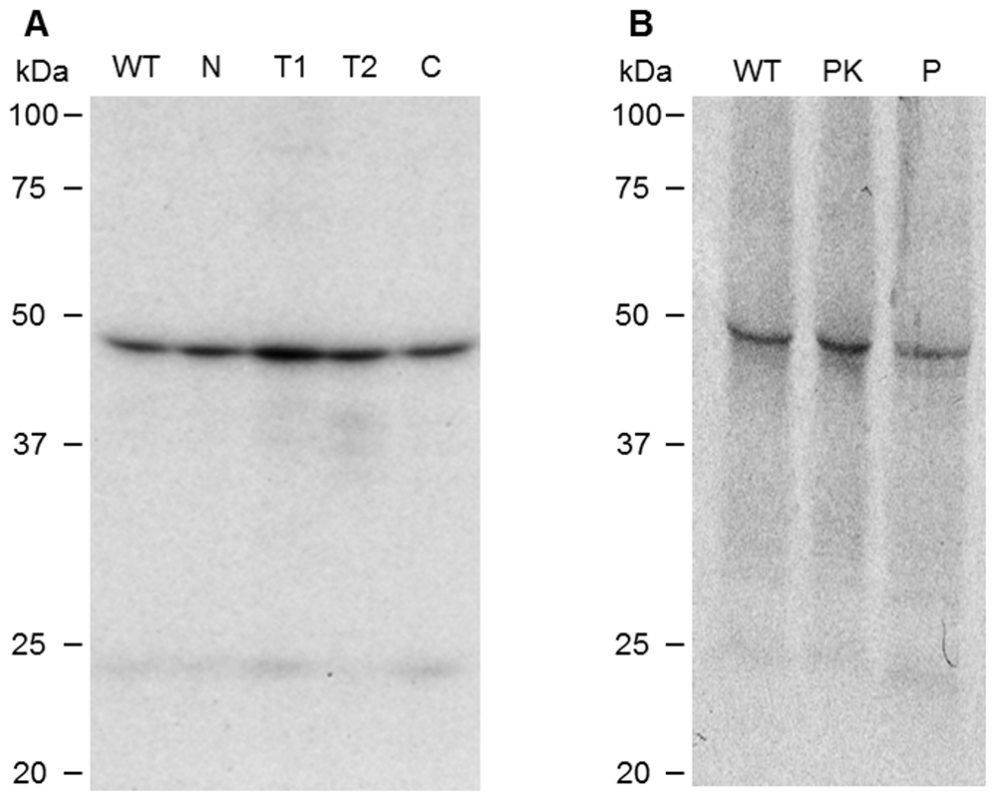
A, BdRap-1 binds to a neuraminidase, trypsin and chymotrypsin-resistant RBC receptor. ^35^S-labelled proteins derived from parasite culture supernatants were mixed with untreated RBCs treated (WT lane), or with RBCs treated with 0.1 U/mL of Neuraminidase (N), 0.1 mg/mL trypsin (T1), 1 mg/mL trypsin (T2), and 1 mg/mL. Separated cells were then lysed and the soluble fraction was immuno-precipitated with anti-rBdRAP-1 antibodies and separated on SDS-PAGE gel. BdRAP-1 appears to be an adhesin that participates in invasion by binding to the RBC surface, as indicated by the presence of a band at the expected size of ∼46 kDa. However, none of the enzyme treatments inhibited or decreased BdRAP-1 binding, as shown the presence of the ∼46 kDa band in all lanes representing treated cells, thus, the binding profile of native BdRAP-1 to the RBC suggests the participation of a novel red cell receptor in merozoite invasion. B, BdRAP-1 binds to a non-proteinacious receptor. Binding profile of BdRAP-1 to untreated RBCs treated (WT lane), or with RBCs treated with 0.5 mg/mL of Proteinase K (PK) or 0.5 mg/mL Papain (P).

Parasite proteins that mediate interaction with host receptors during invasion are commonly localized in the apical organelles. Having established its location at the invasive apical end of the merozoite and homology with other known *Babesia* RAP-1 proteins, we performed assays to determine whether native BdRAP-1 binds to RBCs. Members of the RAP-1 protein family in *Babesia* are involved during the complex process of invasion of host RBCs through direct binding with receptor(s) on the host-cell surface [1], [30]. Identifying both the parasite ligands and their complementary RBC receptor in each part of the invasion process is critical to understanding the invasion biology of *B. divergens* in the human host, and provides continuing information for the development of effective intervention and prevention strategies. Studies have shown that extracellular merozoites release parasite proteins into the culture, and such culture supernatants can be a source of parasite ligands that bind erythrocytes. Thus, [^35^S]methionine/cysteine-labeled spent merozoite supernatants were used as the source of RBC-binding proteins, and when the eluate was immunoprecipitated with anti-rBdRAP-1 antiserum, a dominant band at ∼46 kDa was seen (Figure 4, Panel A, lane WT). Thus, BdRAP-1 appears to be an adhesin that participates in invasion by binding to the RBC surface. Treatment of erythrocytes with enzymes that selectively cleave moieties of surface proteins, followed by an analysis of the resulting effects on BdRAP-1 binding, afforded a first indication of RBC molecules that could serve as receptor(s) for BdRAP-1 during invasion. RBCs were treated with neuraminidase, trypsin and chymotrypsin, and used for binding assays with radio-labeled *B. divergens* parasite supernatant in several independent experiments to detect the receptor recognized by native BdRAP-1. Figure 4 shows the RBC receptor profile for BdRAP-1. None of the enzyme treatments inhibited or decreased BdRAP-1 binding. Thus, the binding profile of native BdRAP-1 to the RBC initially suggests the participation of a novel red cell receptor in merozoite invasion.

### BdRAP-1 binds to erythrocytes via non-proteinacious receptor

To further investigate if the receptor was composed of protein binding assays were carried out on RBCs that were treated with proteinase K and papain, treatments which have been shown to denude the red cell of almost all surface protein. Surprisingly, BdRAP-1 was found to bind these treated RBCs indicating that the RBC moieties involved in binding are not protein and may well be composed of lipid. Future work may elucidate the precise nature of this interaction.

### BdRAP-1 appears to be an immuno-dominant antigen recognized by infected bovine and gerbil sera

**Figure 5:**
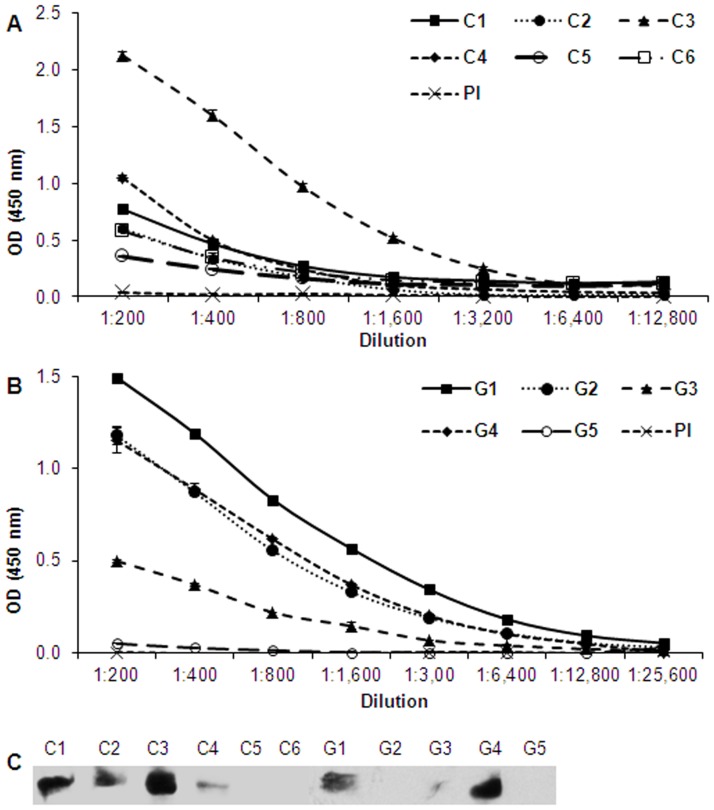
Antigenicity of recombinant BdRAP-1 against sera from *B. divergens* –infected cows and hamsters. A, The reactivity of rBdRAP-1 in ELISA with pre-immune (PI) sera and experimentally *B. divergens*-infected sera from cows (n = 6). All infected bovine sera showed higher OD values than non-infected sera in a dilution-dependant manner at all dilutions. B, The reactivity of rBdRAP-1 in ELISA with pre-immune (PI) sera and experimentally *B. divergens*-infected sera from gerbils (n = 5). Four of the five gerbil sera showed s higher OD values than non-infected sera in a dilution-dependant manner at dilutions 1∶200 to 1∶6,400. One gerbil serum (G5) showed no reactivity. At dilutions 1∶12,800 and 1∶26,600, there is no difference in the reactivity of the gerbil sera and the pre-immune sera. C, Specificity for the animal serum against recombinant BdRAP-1 was confirmed independently by immunoblotting. Four of the six bovine sera and two of the three gerbil sera reacted strongly against rBdRAP-1. One gerbil sera (G3) reacted weakly. Sera which showed the greatest reactivity in ELISA (C3, G1 and G4) also showed greatest reactivity on the blots. Sera G2 and G3 showed moderate reactivity in ELISA but surprisingly very low reactivity in the Western analysis. Sera which did not show any significant reactivity in the ELISA (C5,C6 and G5) also did not react against rBdRAP-1 by immune-blotting.

Sera from six cows and five gerbils infected with *B. divergens* was assayed for reactivity against the rBdRAP-1 using an enzyme-linked immunosorbent assay (ELISA). Positive reactivity was defined as a reading of greater than twofold the values obtained for the negative control samples (pre-immune from the same animals). The highest bovine serum responder, C3, reacted at 37-fold higher, and the lowest responder, C5, reacted at 14-fold higher than the negative control, respectively, at 1∶200 dilution. Further, the samples that showed reactivity remained significantly higher than the negative control reactivity at dilutions of 1∶1,600–1∶12,800 (Figure 5A). A similar pattern of reactivity was observed when the infected gerbil sera was used to assess antibodies to BdRAP-1, with the highest gerbil serum, G4, showing >700-fold reactivity against the negative control at 1∶200 dilution (Figure 5B). One of the gerbil sera (G5) did not react at any dilution, and the reactivity observed for the remaining four gerbil sera were not significantly higher than the negative control at dilutions 1∶6,400 to 1∶25,600. To confirm that these sera are specifically recognizing BdRAP-1, a confirmatory Western Blot was run using purified BdRAP-1. As can be seen from Figure 5C, the animals that gave the highest reactivity on ELISA assays (C3, G1and G4), also recognized rBdRAP-1 on the blots. On the whole, sera which did not show any significant reactivity in the ELISA (C5,C6 and G5) also did not react against rBdRAP-1 by immune-blotting. However, sera from gerbils G2 and G3, which showed reactivity in the ELISA did not show reactivity during immunoblotting. This discrepancy may be due to the denaturation of immuno-reactive epitopes during SDS-PAGE. The high level of reactivity of anti-BdRAP-1 antibodies within these sera suggest that the BdRAP-1 protein is highly immunogenic and suggests it could form the basis of a diagnostic tool. This would be particularly useful in transfusion medicine where there is currently no licensed screening assay for the detection of *Babesia* parasites. Recipients of blood products are at greater risk of developing severe babesiosis due to factors such as extremes in age, lack of a spleen, hemoglobinopathies, cancers, HIV, and use of immunosuppressive therapy [31], yet the donor population largely consists of healthy, asymptomatic adults. The high reactivity also suggests that BdRAP-1 might be a potential vaccine candidate.

### Anti-BdRAP-1 antibodies do not significantly inhibit invasion *in vitro*

**Figure 6:**
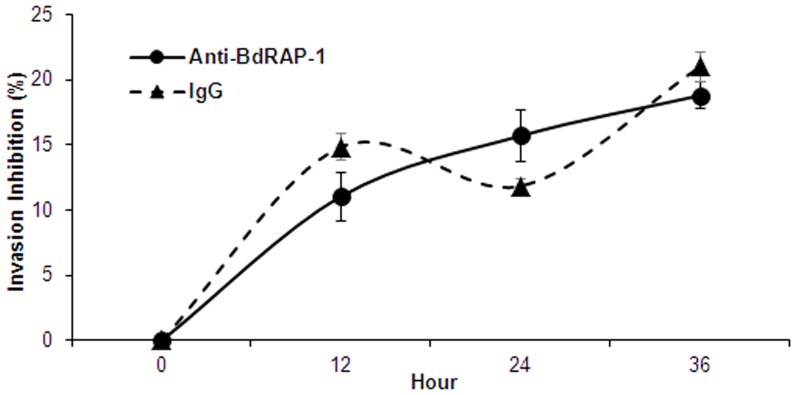
Antibodies against BdRAP-1 do not significantly inhibit parasite invasion *in vitro*. *B. divergens* cultures in human cells were maintained for 36 h in the presence of 2 mg/mL of the purified IgG fraction of anti-rBdRAP-1 antibodies from rabbit serum or in the presence of the equivalent concentration of purified rabbit pre-immune IgG. The level of growth inhibition compared to a no-serum control was determined every 12 h. The presence of BdRAP-1 (indicated by a solid line) was able to inhibit growth of *B. divergens* by 11% and 16% at 12 h and 24 h, respectively, to a maximum inhibition of 19% at 36 h. however, this is not significantly different from the inhibition due to the presence of pre-immune IgG (indicated by a dashed line), which inhibited growth by 20% at 36 h, and the lack of inhibition specifically due to anti-BdRAP-1 antibodies suggests multiple alternative pathways of invasion are available to the parasite, or the role of this ligand is not restricted to invasion only.

Purified IgG from the rabbit anti-rBdRAP-1 serum was used to evaluate the role of native BdRAP-1 during the invasion process. Cultures were established with a starting parasitaemia of ∼4% and purified anti-rBdRAP-1 IgG was added to a final concentration of 2 mg/mL to the cultures at 0 h and refreshed at 24 h to the same concentration. Smears were made and read at 0 h, 12 h, 24 h, and 36 h. Purified rabbit IgG from pre-immune serum was used at the same concentration as a control. The growth inhibition observed in the presence of anti-BdRAP-1 antibodies after 12 h was 11% (Figure 6), and the maximum inhibition observed was 19% after 36 h. However, there was no significant difference in the growth inhibition observed for the pre-immune serum, which showed 14% and 21% inhibition at 12 h and 36 h, respectively. The limited growth inhibition observed suggests the binding of BdRAP-1 to the RBC receptor during invasion is not the primary pathway this species uses to invade host cells, or,, there is significant redundancy in the invasion mechanisms and multiple invasion pathways are available to the parasite. Additionally, the full repertoire of RAP-1 and other RAP-1 related molecules in the parasite genome has not been accounted for, as it has in other *Babesia* species [32]–[35], and thus, homologs of this antigen may serve in its role when BdRAP-1 is neutralized by antibodies. It seems increasingly likely that RAP-1 in malaria may not directly play a role in the invasive process, but like other proteins housed in the rhoptry bulb, may participate in events downstream of active entry, like enhancing parasite survival in the host cell [36]–[38]. Thus, binding of BdRAP-1 to RBCs may not reflect an *in vivo* role for the ligand but relate to potential interactions that would occur between RAP-1 and RBC proteins transferred to the parasitophorous vacuole membrane (PVM) during invasion, or maybe the result of non-specific binding of the ligand to lipid membranes, including both the RBC surface and the PVM. Future experiments directed to the dissection of this interaction may help elucidate the true role of these rhoptry proteins.

### Conclusions

Although other *Babesia* RAP-1 proteins have been identified [19], [26], [27], [39], [40], we present the gene encoding a RAP-1 homologue of a human babesia parasite BdRouen1987, the ∼46 kDa *Babesia divergens* RAP-1 protein and have characterized this important antigen. BdRAP-1 is located in the bulb of the rhoptries and binds to an RBC receptor(s) that is resistant to sialidases and various proteinases. We do not find significant reduction in growth with anti-rRAP1 *in vitro*, suggesting the presence of alternative pathways of invasion or alternative roles for this ligand. Identification and assessment of the antigens used for RBC invasion and remodelling of RBC after invasion are essential for understanding the invasion biology of *B. divergens* to determine the most effective interventions.

## References

[1] Yokoyama N, Okamura M, Igarashi I (2006) Erythrocyte invasion by Babesia parasites: current advances in the elucidation of the molecular interactions between the protozoan ligands and host receptors in the invasion stage. Vet Parasitol 138: 22–32. 16504403 10.1016/j.vetpar.2006.01.037

[2] Sibley LD (2004) Intracellular parasite invasion strategies. Science 304: 248–253. 15073368 10.1126/science.1094717

[3] Sam-Yellowe TY (1996) Rhoptry organelles of the apicomplexa: Their role in host cell invasion and intracellular survival. Parasitol Today 12: 308–316. 15275182 10.1016/0169-4758(96)10030-2

[4] Besteiro S, Dubremetz JF, Lebrun M (2011) The moving junction of apicomplexan parasites: a key structure for invasion. Cell Microbiol 13: 797–805. 21535344 10.1111/j.1462-5822.2011.01597.x

[5] Spielman A, Wilson ML, Levine JF, Piesman J (1985) Ecology of Ixodes dammini-borne human babesiosis and Lyme disease. Annu Rev Entomol 30: 439–460. 3882050 10.1146/annurev.en.30.010185.002255

[6] Lantos PM, Krause PJ (2002) Babesiosis: similar to malaria but different. Pediatr Ann 31: 192–197. 11905293 10.3928/0090-4481-20020301-10

[7] Kjemtrup AM, Conrad PA (2000) Human babesiosis: an emerging tick-borne disease. Int J Parasitol 30: 1323–1337. 11113258 10.1016/s0020-7519(00)00137-5

[8] Vannier E, Krause PJ (2009) Update on babesiosis. Interdiscip Perspect Infect Dis 2009: 984568. 19727410 10.1155/2009/984568 PMC2734943

[9] Gray J, Zintl A, Hildebrandt A, Hunfeld KP, Weiss L (2010) Zoonotic babesiosis: overview of the disease and novel aspects of pathogen identity. Ticks Tick Borne Dis 1: 3–10. 21771506 10.1016/j.ttbdis.2009.11.003

[10] Lobo CA, Cursino-Santos JR, Alhassan A, Rodrigues M (2013) Babesia: an emerging infectious threat in transfusion medicine. PLoS Pathog 9: e1003387. 23853577 10.1371/journal.ppat.1003387 PMC3708872

[11] Homer MJ, Aguilar-Delfin I, Telford SR 3rd, Krause PJ, Persing DH (2000) Babesiosis. Clin Microbiol Rev 13: 451–469. 10885987 10.1128/cmr.13.3.451-469.2000 PMC88943

[12] Garnham PC (1980) Human babesiosis: European aspects. Trans R Soc Trop Med Hyg 74: 153–155. 6770500 10.1016/0035-9203(80)90232-1

[13] Zintl A, Mulcahy G, Skerrett HE, Taylor SM, Gray JS (2003) Babesia divergens, a bovine blood parasite of veterinary and zoonotic importance. Clin Microbiol Rev 16: 622–636. 14557289 10.1128/CMR.16.4.622-636.2003 PMC207107

[14] Dammin GJ, Spielman A, Benach JL, Piesman J (1981) The rising incidence of clinical Babesia microti infection. Hum Pathol 12: 398–400. 7195871 10.1016/s0046-8177(81)80020-2

[15] Ruebush TK 2nd, Juranek DD, Chisholm ES, Snow PC, Healy GR, et al (1977) Human babesiosis on Nantucket Island. Evidence for self-limited and subclinical infections. N Engl J Med 297: 825–827. 561308 10.1056/NEJM197710132971511

[16] Kats LM, Black CG, Proellocks NI, Coppel RL (2006) Plasmodium rhoptries: how things went pear-shaped. Trends Parasitol 22: 269–276. 16635585 10.1016/j.pt.2006.04.001

[17] Preiser P, Kaviratne M, Khan S, Bannister L, Jarra W (2000) The apical organelles of malaria merozoites: host cell selection, invasion, host immunity and immune evasion. Microbes Infect 2: 1461–1477. 11099933 10.1016/s1286-4579(00)01301-0

[18] Kaneko O (2007) Erythrocyte invasion: vocabulary and grammar of the Plasmodium rhoptry. Parasitol Int 56: 255–262. 17596999 10.1016/j.parint.2007.05.003

[19] Skuce PJ, Mallon TR, Taylor SM (1996) Molecular cloning of a putative rhoptry associated protein homologue from Babesia divergens. Mol Biochem Parasitol 77: 99–102. 8784776 10.1016/0166-6851(96)02570-4

[20] Gorenflot A, Brasseur P, Precigout E, L'Hostis M, Marchand A, et al (1991) Cytological and immunological responses to Babesia divergens in different hosts: ox, gerbil, man. Parasitol Res 77: 3–12. 1994368 10.1007/BF00934377

[21] Lobo CA (2005) Babesia divergens and Plasmodium falciparum use common receptors, glycophorins A and B, to invade the human red blood cell. Infect Immun 73: 649–651. 15618210 10.1128/IAI.73.1.649-651.2005 PMC538995

[22] Florin-Christensen M, Suarez CE, Hines SA, Palmer GH, Brown WC, et al (2002) The Babesia bovis merozoite surface antigen 2 locus contains four tandemly arranged and expressed genes encoding immunologically distinct proteins. Infect Immun 70: 3566–3575. 12065497 10.1128/IAI.70.7.3566-3575.2002 PMC128111

[23] Suarez CE, Florin-Christensen M, Hines SA, Palmer GH, Brown WC, et al (2000) Characterization of allelic variation in the Babesia bovis merozoite surface antigen 1 (MSA-1) locus and identification of a cross-reactive inhibition-sensitive MSA-1 epitope. Infect Immun 68: 6865–6870. 11083806 10.1128/iai.68.12.6865-6870.2000 PMC97791

[24] Kania SA, Allred DR, Barbet AF (1995) Babesia bigemina: host factors affecting the invasion of erythrocytes. Exp Parasitol 80: 76–84. 7821413 10.1006/expr.1995.1009

[25] Wittner M, Rowin KS, Tanowitz HB, Hobbs JF, Saltzman S, et al (1982) Successful chemotherapy of transfusion babesiosis. Ann Intern Med 96: 601–604. 7200341 10.7326/0003-4819-96-5-601

[26] Dalrymple BP, Casu RE, Peters JM, Dimmock CM, Gale KR, et al (1993) Characterisation of a family of multi-copy genes encoding rhoptry protein homologues in Babesia bovis, Babesia ovis and Babesia canis. Mol Biochem Parasitol 57: 181–192. 8433711 10.1016/0166-6851(93)90194-3

[27] Suarez CE, Palmer GH, Jasmer DP, Hines SA, Perryman LE, et al (1991) Characterization of the gene encoding a 60-kilodalton Babesia bovis merozoite protein with conserved and surface exposed epitopes. Mol Biochem Parasitol 46: 45–52. 1712911 10.1016/0166-6851(91)90197-e

[28] Howard RF, Reese RT (1990) Plasmodium falciparum: hetero-oligomeric complexes of rhoptry polypeptides. Exp Parasitol 71: 330–342. 1698657 10.1016/0014-4894(90)90038-e

[29] Howard RF, Narum DL, Blackman M, Thurman J (1998) Analysis of the processing of Plasmodium falciparum rhoptry-associated protein 1 and localization of Pr86 to schizont rhoptries and p67 to free merozoites. Mol Biochem Parasitol 92: 111–122. 9574915 10.1016/s0166-6851(97)00238-7

[30] Yokoyama N, Suthisak B, Hirata H, Matsuo T, Inoue N, et al (2002) Cellular localization of Babesia bovis merozoite rhoptry-associated protein 1 and its erythrocyte-binding activity. Infect Immun 70: 5822–5826. 12228313 10.1128/IAI.70.10.5822-5826.2002 PMC128354

[31] Gubernot DM, Lucey CT, Lee KC, Conley GB, Holness LG, et al (2009) Babesia infection through blood transfusions: reports received by the US Food and Drug Administration, 1997–2007. Clin Infect Dis 48: 25–30. 19035776 10.1086/595010

[32] Suarez CE, Palmer GH, Florin-Christensen M, Hines SA, Hotzel I, et al (2003) Organization, transcription, and expression of rhoptry associated protein genes in the Babesia bigemina rap-1 locus. Mol Biochem Parasitol 127: 101–112. 12672519 10.1016/s0166-6851(02)00311-0

[33] Hotzel I, Suarez CE, McElwain TF, Palmer GH (1997) Genetic variation in the dimorphic regions of RAP-1 genes and rap-1 loci of Babesia bigemina. Mol Biochem Parasitol 90: 479–489. 9476795 10.1016/s0166-6851(97)00182-5

[34] Mishra VS, McElwain TF, Dame JB, Stephens EB (1992) Isolation, sequence and differential expression of the p58 gene family of Babesia bigemina. Mol Biochem Parasitol 53: 149–158. 1501634 10.1016/0166-6851(92)90017-e

[35] Suarez CE, Laughery JM, Bastos RG, Johnson WC, Norimine J, et al (2011) A novel neutralization sensitive and subdominant RAP-1-related antigen (RRA) is expressed by Babesia bovis merozoites. Parasitology 138: 809–818. 21554842 10.1017/S0031182011000321

[36] Ling IT, Florens L, Dluzewski AR, Kaneko O, Grainger M, et al (2004) The Plasmodium falciparum clag9 gene encodes a rhoptry protein that is transferred to the host erythrocyte upon invasion. Mol Microbiol 52: 107–118. 15049814 10.1111/j.1365-2958.2003.03969.x

[37] Nguitragool W, Bokhari AA, Pillai AD, Rayavara K, Sharma P, et al (2011) Malaria parasite clag3 genes determine channel-mediated nutrient uptake by infected red blood cells. Cell 145: 665–677. 21620134 10.1016/j.cell.2011.05.002 PMC3105333

[38] Riglar DT, Richard D, Wilson DW, Boyle MJ, Dekiwadia C, et al (2011) Super-resolution dissection of coordinated events during malaria parasite invasion of the human erythrocyte. Cell Host Microbe 9: 9–20. 21238943 10.1016/j.chom.2010.12.003

[39] Machado RZ, McElwain TF, Suarez CE, Hines SA, Palmer GH (1993) Babesia bigemina: isolation and characterization of merozoite rhoptries. Exp Parasitol 77: 315–325. 8224087 10.1006/expr.1993.1089

[40] Ikadai H, Xuan X, Igarashi I, Tanaka S, Kanemaru T, et al (1999) Cloning and expression of a 48-kilodalton Babesia caballi merozoite rhoptry protein and potential use of the recombinant antigen in an enzyme-linked immunosorbent assay. J Clin Microbiol 37: 3475–3480. 10523537 10.1128/jcm.37.11.3475-3480.1999 PMC85671

